# Using Hidden Markov Models to Improve Quantifying Physical Activity in Accelerometer Data – A Simulation Study

**DOI:** 10.1371/journal.pone.0114089

**Published:** 2014-12-02

**Authors:** Vitali Witowski, Ronja Foraita, Yannis Pitsiladis, Iris Pigeot, Norman Wirsik

**Affiliations:** 1 Department Biometry and Data Management, Leibniz Institute for Prevention Research and Epidemiology – BIPS, Bremen, Germany; 2 Department of Mathematics and Computer Science, University of Bremen, Bremen, Germany; 3 School of Sport and Service Management, University of Brighton, Eastbourne, United Kingdom; Université de Nantes, France

## Abstract

**Introduction:**

The use of accelerometers to objectively measure physical activity (PA) has become the most preferred method of choice in recent years. Traditionally, cutpoints are used to assign impulse counts recorded by the devices to sedentary and activity ranges. Here, hidden Markov models (HMM) are used to improve the cutpoint method to achieve a more accurate identification of the sequence of modes of PA.

**Methods:**

1,000 days of labeled accelerometer data have been simulated. For the simulated data the actual sedentary behavior and activity range of each count is known. The cutpoint method is compared with HMMs based on the Poisson distribution (HMM[Pois]), the generalized Poisson distribution (HMM[GenPois]) and the Gaussian distribution (HMM[Gauss]) with regard to misclassification rate (MCR), bout detection, detection of the number of activities performed during the day and runtime.

**Results:**

The cutpoint method had a misclassification rate (MCR) of 11% followed by HMM[Pois] with 8%, HMM[GenPois] with 3% and HMM[Gauss] having the best MCR with less than 2%. HMM[Gauss] detected the correct number of bouts in 12.8% of the days, HMM[GenPois] in 16.1%, HMM[Pois] and the cutpoint method in none. HMM[GenPois] identified the correct number of activities in 61.3% of the days, whereas HMM[Gauss] only in 26.8%. HMM[Pois] did not identify the correct number at all and seemed to overestimate the number of activities. Runtime varied between 0.01 seconds (cutpoint), 2.0 minutes (HMM[Gauss]) and 14.2 minutes (HMM[GenPois]).

**Conclusions:**

Using simulated data, HMM-based methods were superior in activity classification when compared to the traditional cutpoint method and seem to be appropriate to model accelerometer data. Of the HMM-based methods, HMM[Gauss] seemed to be the most appropriate choice to assess real-life accelerometer data.

## Introduction

Currently physical inactivity is considered a major risk factor for several health disorders like cancer [1], obesity [2], cardiovascular disorders [3], muscular skeletal disorders [4], as well as mental disorders [5]. An appropriate assessment of physical activity (PA) is therefore essential in disciplines like medicine and epidemiology to improve the existing evidence-base. The use of accelerometers as an objective measurement of PA has become the most preferred method of choice in recent years, as modern devices allow high frequency measurements for extended periods of time. These now relatively inexpensive devices collect information known as (impulse-)*counts* and provide information on intensity and duration of PA in an individual.

Counts represent a device-specific numeric quantity which is generated by an accelerometer for a specific time unit (*epoch*) (e.g. 1 to 60 sec). This quantity is proportional to the intensity of the physical activity performed by the subject. The sequence of activities during a day is stored as a time series of counts by the device. The most common approach to derive the pattern of PA and its energy expenditure is to map these counts to a certain number of sedentary and activity ranges, such as sedentary, light, moderate and vigorous activity. The duration of PA within the same activity range is known as *bout* and can be easily extracted from a given sequence of counts. A bout is defined as the time period in which the subject remains within one activity range without changing to another. Activity ranges are separated by thresholds known as *cutpoints*. Cutpoints for different age groups are available for children [6], [7], [8], [9], [10], [11], [12] and adults [13], [14], [15] allowing to assess the overall time spent in these ranges of PA.

**Figure 1:**
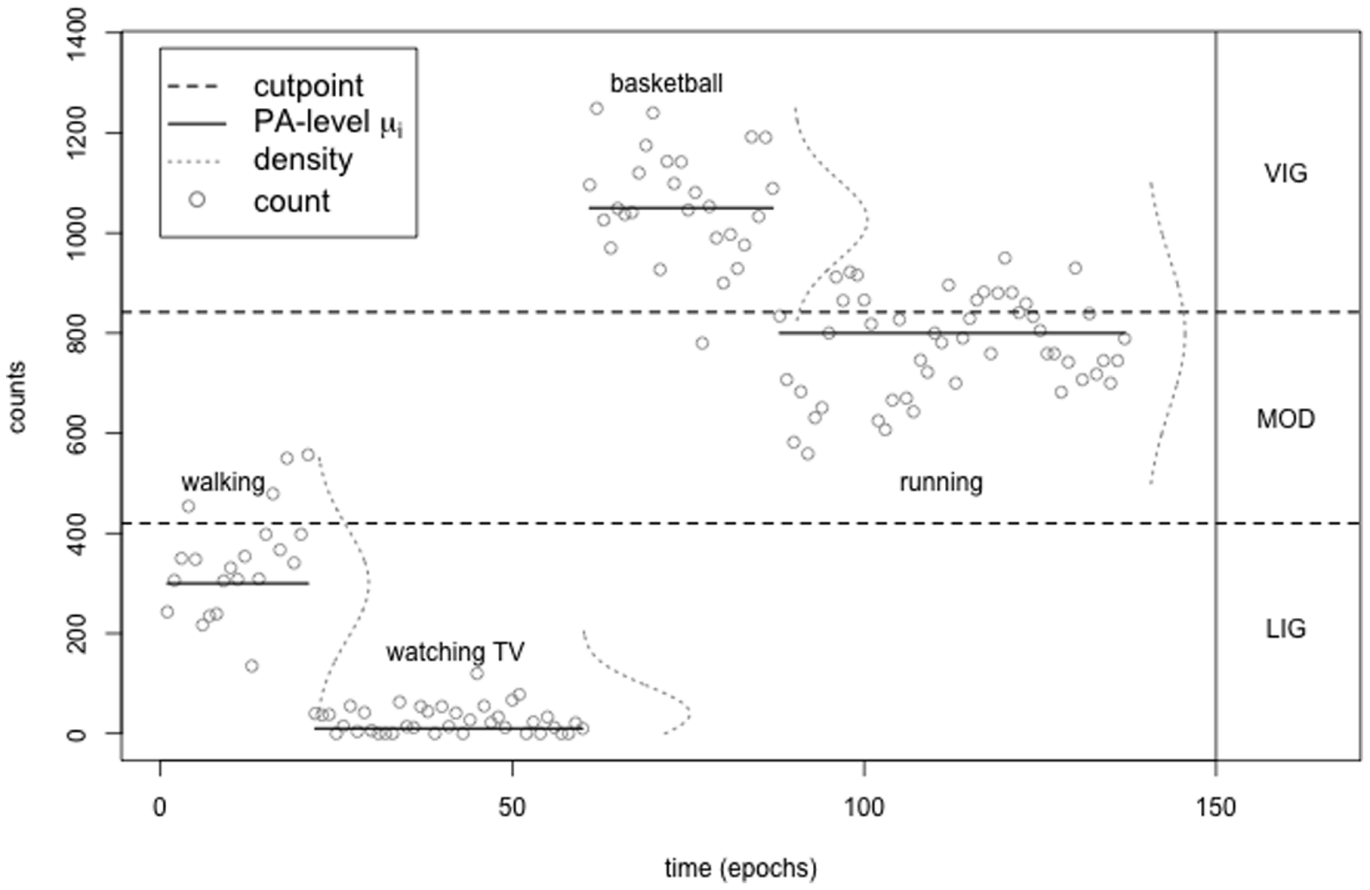
Modeling of accelerometer counts using HMMs. The figure shows the three activity ranges LIG, MOD, VIG, separated by the cutpoints at 420 counts and 842 counts. The accelerometer counts 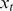 scatter around four different activity states (“watching TV”, “walking”, “running” and “playing basketball”) following a state dependent distribution 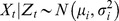 with 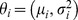 and fictitious PA-levels 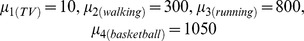 respectively.

While the ease of implementation of this *cutpoint method* is an obvious advantage, this method has certain important disadvantages. Counts are being incorrectly assigned to the wrong activity range, leading to misclassification and thereby to an increase of bouts. In the following we assume that the PA of an individual is composed of a sequence of non-overlapping bouts, i.e. each bout being a discrete activity, which is performed over a period of time. Furthermore, the modes of activity can be represented by a ‘true’ average count level. This assumption is depicted in Figure 1. The person first takes a short walk, after which she/he watches TV, followed by a game of basketball and running afterwards. The solid black lines represent the ‘true’ average count level for each of these activities. For example the short walk at the beginning has a true count level of about 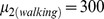 counts per epoch, which can be understood as the true intensity level of this walk. The counts registered by the accelerometer scatter around this true level, following a certain distribution (dotted grey line). So the PA depicted in Figure 1 consists of four separate bouts, with four distinct PA-levels 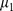 to 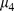.

As long as the variation around the true intensity level is small and the true level is not close to a cutpoint the complete mode of activity can be correctly assigned to its corresponding activity range. However, in real-life applications the variation of counts and the resulting scattering is large, leading to considerable misclassification of the registered counts into erroneous activity ranges. As a consequence the number of bouts is dramatically increased, as a subject seems to switch from one activity range to another and back again within a few epochs. Therefore, the duration a subject spends in one activity range can be significantly under- or overestimated (cf. numerous validation studies performed to date e.g. [16], [17], [18], [19]).

There has been a number of attempts to resolve the misclassification issue. For example, Pober et al. [20] proposed stochastic models to allow the identification of modes of activity like working at a computer, walking, walking uphill and vacuuming in accelerometer data. A *hidden Markov model* (HMM) was successfully trained to identify these activities. The model correctly identified the activity mode in 80.8% of the data. Vacuuming was correctly identified most frequently in 98.8% of all cases, and walking/walking uphill in about 62%. This approach requires annotated data for training the HMM and activity mode identification is therefore limited to the modes used during training. In order to use this method in free living environments, one would need to train the HMM with all possible activity modes.

As a solution to the misclassification problem caused by large variation of the counts registered by accelerometers, we suggest a new approach that combines the HMM-based method with the traditional cutpoint method. The aim is to provide a better estimate of the activity modes that generated the sequence of counts during the day and by this to decrease the misclassification error, which is inevitably introduced by the cutpoint approach. In order to achieve this, an HMM-based approach was developed to model accelerometer data. 1,000 days of labeled accelerometer data were simulated. HMM models based on the Gaussian, the Poisson and the generalized Poisson distribution were compared with the cutpoint method with regard to misclassification rate (MCR), bout detection, detection of the number of activities performed during the day and runtime.

## Methods

### Traditional cutpoint approach

The cutpoint method assigns an activity range to each epoch. There are various cutpoints available in the literature. Any of these cutpoints could have been used for our simulation study where we decided to use cutpoints from Pate et al. [9]. According to [9] epochs with <420 counts/15 sec are assigned to light physical activity (*LIG*) with 0–3 *metabolic equivalent of task* (*METs*), epochs with 420–841 counts/15 sec to moderate physical activity (*MOD*) with 3–6 METs and epochs >841 counts/15 sec to vigorous physical activity (*VIG*) with more than 6 METs.

### HMM-based approach

An HMM is a stochastic model based on the idea that an observed time series has been generated by an underlying unobservable, time and value discrete, stochastic process whose random variables 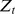 are hidden. This sequence of hidden states satisfies the Markov property and forms a Markov chain, i.e. the transition probability to switch from one state to another only depends on the state of interest and is independent of all states prior to *t*. The hidden Markov chain represents a sequence of unobserved random variables 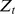 with a finite number of states 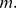 Let 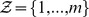 denote the set of possible states and represent the realization of 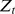 at point in time 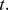 Each state 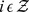 symbolizes different activities that change from one activity 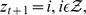 to another 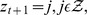 over time.

The states, however, cannot be observed directly, but they generate a state-dependent output according to a known or presumed probability distribution (see [21], [22] for further details on HMMs). For the purpose of this analysis, the hidden sequence of states is the true, but unknown sequence of PA each subject performed in a free living-environment, while the recorded accelerometer counts 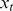 are the observed realizations of random variables 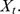 The sequence of hidden states satisfies the *Markov property*:
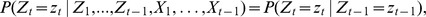
i.e. each activity solely depends on its predecessor. The resulting time series of length 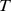
of performed physical activities 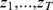 can only be observed indirectly via a parallel time series represented by the assessed accelerometer counts.

The probability of a Markov chain to switch from state 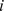 to state 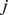 is given by the *transition probability 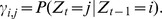
* A Markov chain is called *homogeneous*, if the transition probability 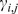 is independent of 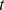 for all pairs of 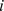 and 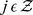. The transition probability of a homogeneous Markov chain with finite 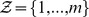 can be summarized in an 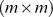 *transition matrix 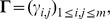
* with elements of 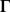 being probabilities and therefore the following conditions have to be fulfilled: and A Markov chain is fully defined by this transition matrix 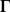 and a vector containing the *initial probabilities* 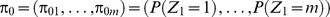 with 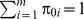 for the first state.

In the HMM-based approach, each state 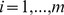 is linked with the mean activity count 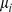 of the PA, which the state represents. 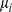 denotes the *PA-level* of the PA 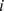. Furthermore, the variable 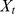 is assumed to be conditionally independent of all remaining variables given its hidden PA
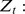


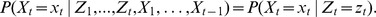



This means, at each point in time 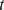, the count 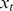 is assumed to be generated by a certain distribution, which depends on the activity state 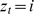, with the corresponding PA-level 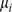 as mean of this distribution. The *observation distribution* is the probability that 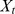 takes a value 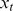 under the condition that 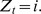 The observation distributions are assumed to be a subset of a whole class of distributions to be specified in advance. Depending on the class of distributions, 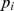 is determined by 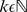 parameters. These form the *parameter vector* 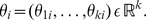 The 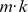 parameters in turn form the matrix 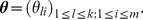 An HMM is fully described by its *model-specific parameter 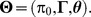
* This setting is illustrated in Figure 1 and depicts the fictitious output of an accelerometer, while the subject performed four activities ‘walking’, ‘watching TV’, ‘running’ and ‘playing basketball’. The sequence of activities is assumed to follow a Markov chain, and the accelerometer counts are assumed to be generated by four activity-state-dependent Gaussian distributions with the corresponding PA-levels 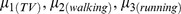 and 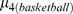 as their means.

The HMM approach developed for such situations can be subdivided into the following three steps.

**Step 1: Building an HMM for an observed time series of counts.** The model specific parameters 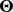 of the HMM given an observed time series of counts 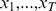 are estimated. This is referred to as *training of the HMM*. Parameter estimation can either be performed by numerical maximization of the likelihood of the model with respect to 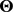 or by utilizing the so-called *Baum-Welch algorithm* [23] which is commonly used to fit HMMs.

Typically the number of hidden states *m* (respectively the number of hidden activities) given the counts 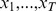 is unknown. In this case the basic idea is to train several HMMs with different numbers of states 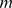 and to evaluate the goodness of fit of the model by the *Bayesian Information Criterion (BIC)* and *Akaike’s Information Criterion (AIC)*. If both criteria suggest a different number of states, then one may opt for fewer states to have a more simplistic model or for a larger number of states if this better reflects the underlying practical situation.

**Step 2: Decoding the hidden sequence of PA-levels.** After the model parameters 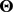 and an appropriate number of physical activities 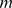 have been estimated, the resulting HMM is used to link each count 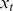 to an estimated PA-level 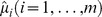.

Step 2.1 First, the *Viterbi algorithm* [24], [25] decodes the globally most likely sequence of hidden activities denoted by 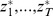 for the trained HMM and the same time series of counts 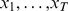 that was used to train the HMM in Step 1 by comparing the joint probability of all *T* hidden states and the observed accelerometer counts. Alternatively, a local method can be used to decode the most likely hidden activity 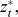 given all accelerometer counts 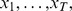 for each single 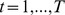 by comparing the joint probability of the hidden state at point in time 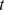 and the observed accelerometer counts.

**Figure 2:**
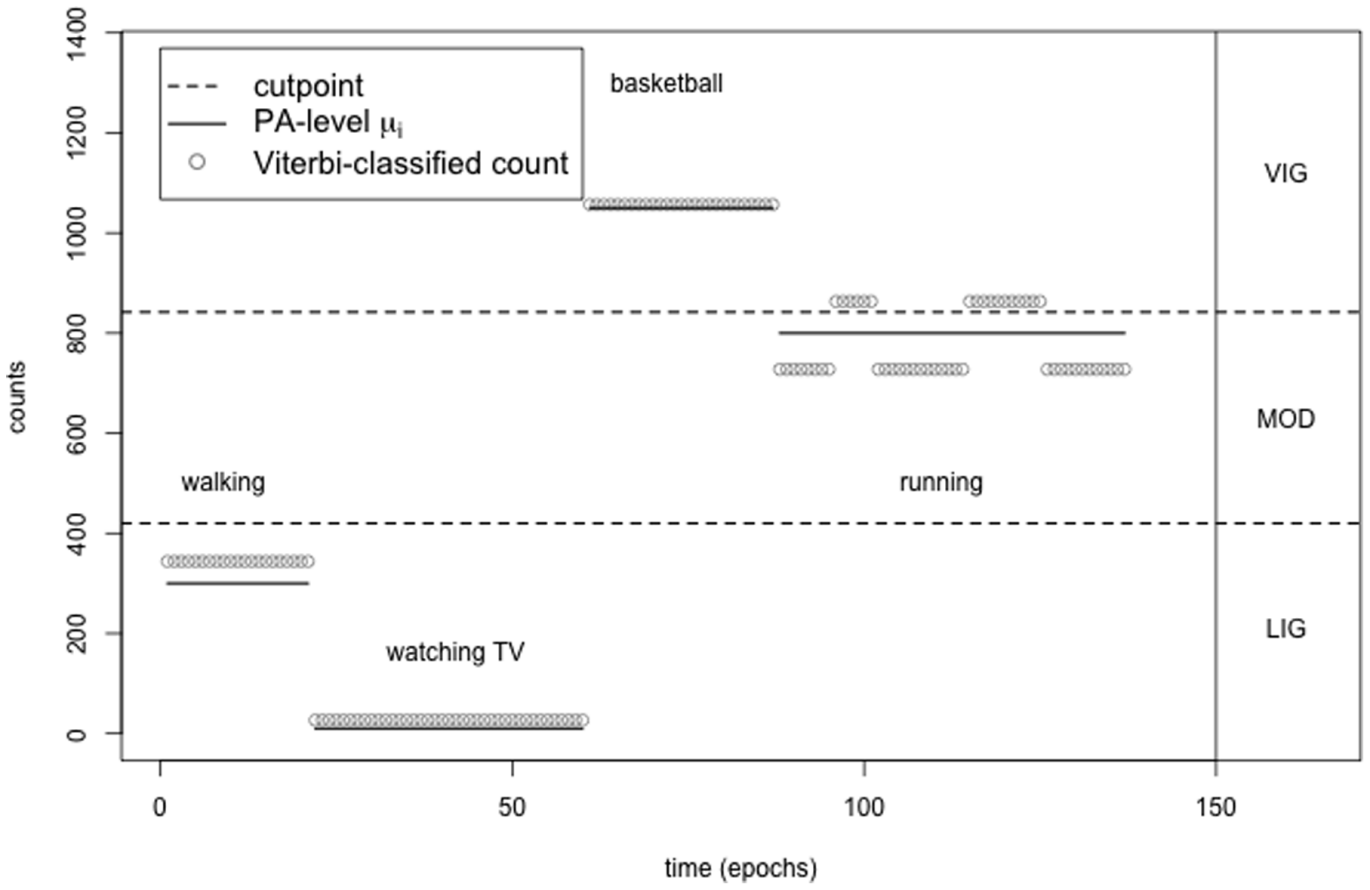
HMM-decoding using the Viterbi algorithm to extract the most likely sequence of physical activity.

Step 2.2 Second, each count 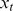 is assigned to the estimated PA-level 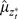 that corresponds to the decoded state 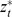 at this point in time. Step 2.2 is demonstrated in Figure 2. In this example, the trained HMM with 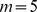 leads to an overfitting of the four activities performed, where the state ‘running’ is mistakenly split into two different PA-levels by the decoding step.

**Step 3: Extension of the cutpoint method.** In the last step of our approach, which combines the HMM-based method with the traditional cutpoint approach, each accelerometer count 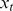 will be assigned via its corresponding (most likely) PA-level 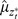 to an activity range 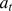, using the traditional cutpoint method.

Overall, the procedure of the new HMM-based cutpoint approach can be summarized as follows:

Step 1: Train the HMM parameters assuming a probability distribution for the counts for each (hidden) PA.

Step 1.1: (optional): Estimate the number of different states *m*.

Step 2: Decode the hidden sequence.

Step 2.1: Estimate the most likely sequence of states (HMM-decoding): 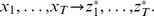


Step 2.2: Assign a PA-level (HMM-decoding): 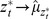


Step 3: Assign an activity range (cutpoint method): 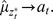


In the example illustrated in Figure 2, the trained HMM identifies five PA-levels 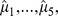 which leads to a misclassification of parts of the state ‘running’ into five instead of one bout, with two bouts being assigned to the highest activity range. Even with this overestimation of five identified PA-levels instead of four, the HMM-based method assigns most counts correctly to their actual activity range. The high number of bouts typically obtained from the cutpoint method is reduced by the HMM-based approach because a Markov chain is assumed to underlie the performed activities at each point in time. The present example consists of three bouts: the first is defined by the two activities ‘walking’ and ‘watching TV’ that correspond to the activity range LIG; the second bout is defined by ‘running’ in MOD and the third by ‘playing basketball’ in VIG. Due to the assumed Markov chain, the HMM-based approach detects eight bouts, which is an overestimation of the true value of three, but results are more precise than those obtained from the traditional cutpoint method, which identifies 25 bouts.

The underlying distributions of the states which generate the observed time series are a priori unknown. In the context of modeling accelerometer counts, three distributions are of particular interest: The first HMM is based on the Poisson distribution, which is typically used to model counts. The second model uses the generalized Poisson distribution [26] that includes a further variance parameter to allow for a larger or smaller variation than the one assumed for a standard Poisson distribution. Real-life accelerometer data typically show larger variability than a simple Poisson distribution can accommodate. For the third HMM, a Gaussian distribution is assumed to capture the random scattering of the counts around the presumed PA-level. For the purpose of the present analysis, the Poisson-based HMM is referred to as HMM[Pois], the HMM based on the generalized Poisson distribution as HMM[GenPois] and the Gaussian-based HMM as HMM[Gauss].

### Simulation Study

The performance of the traditional cutpoint method is assessed by comparing it with the extended cutpoint method using HMMs in terms of (1) the misclassification rate (MCR), calculated as the percentage of how many of the counts were assigned incorrectly to any other activity range than their true activity range, (2) number of bouts correctly identified, (3) number of activity levels correctly identified, and (4) runtime. For this purpose, *labeled* accelerometer count time series, for which the correct activity range of each count is known, with the length of 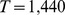 and an epoch length of 15 seconds have been simulated. This particular epoch length and length of 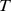 were chosen to reflect typical situations in population-based epidemiological studies [27]. The HMMs does also work with shorter epoch lengths and larger 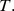 Please note that for our simulated, labeled data, the true sequence of activities and therefore the actual PA-level and also the activity range of each count are known. In total, 1,000 different time series were simulated, each representing 6 hours of counts per day (data available under doi:10.5061/dryad.tq0gt). Counts per day were randomly generated using the negative binomial distribution (with parameters 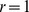 and 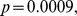 resulting in the lowest PA-level 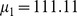) and the Gaussian distribution (with the parameters and 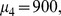 with 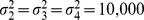) around three or four pre-defined PA-levels (depending on the random time series generated by a Markov chain), with the lowest PA-level (400) chosen to be very close to the lower cutpoint of 420. To create random activity modes that are time periods spent on the same PA-level, e.g. walking or running, the sequence of PA-levels has been generated using a Markov chain. The simulations were designed to reflect free living-environment observations obtained for children (see Table 1). The simulated data were specifically designed to accommodate cutpoints proposed by [9]. As a large amount of misclassification is expected to occur in activity modes close to a cutpoint, the lowest PA-level (400) was intentionally chosen to be close to one cutpoint, in order to demonstrate the advantage of this method. Any other cutpoints available in the literature could have been chosen, since the application of HMMs does not depend on the choice of the cutpoints. On average, one 6 hour day comprised of 23.66 bouts and 3.97 activities during the day. For data simulation and analysis the R package *HMMpa* [28] was used.

**Table 1:** Statistical characteristics of the simulated 1,000 data sets (SD = standard deviation).

	Mean	SD	Min	Median	Max
b [bouts]	23.66	7.03	5.00	23.00	47.00
*m* [activities]	3.97	0.17	3.00	4.00	4.00

## Results

Table 2 displays the MCR of all considered methods based on 1,000 simulated days. The cutpoint method shows the highest misclassification rate with about 11% followed by HMM[Pois] with about 8%. HMM[GenPois] and HMM[Gauss] correctly assign 97% and 98% of all counts to their true activity range, respectively. HMM[GenPois] and HMM[Gauss] outcomes are very close to the simulated data of 23.7 bouts with a mean of 31.2 and 32.5 detected bouts, respectively, while HMM[Pois] detects five times and the cutpoint method ten times as many bouts (Table 2). HMM[Gauss] detects the correct number of bouts in 12.8% and HMM[GenPois] in 16.1% of the days.

**Table 2:** Misclassification rate, number of identified bouts and identified activities for the traditional cutpoint method and the HMM-based method with different state-dependent observation distributions (SD = standard deviation).

Measure	Method	Mean	SD	Min	Median	Max	Correctly identified [%]
**Misclassification rate**	Cutpoint	11.14	2.16	5.35	11.18	19.31	88.86
	HMM[Gauss]	1.77	3.53	0.00	0.90	31.94	98.23
	HMM[Pois]	8.21	5.97	1.53	5.56	32.64	91.79
	HMM[GenPois]	3.03	5.58	0.14	1.18	23.06	96.97
**Number of identified bouts**	Cutpoint	229.55	38.52	129.00	229.00	345.00	0.00
	HMM[Gauss]	32.52	12.84	1.00	31.00	125.00	12.8
	HMM[Pois]	136.43	46.75	37.00	131.00	283.00	0.00
	HMM[GenPois]	31.16	9.86	13.00	31.00	51.00	16.1
**Number of identified activities**	Cutpoint	–	–	–	–	–	–
	HMM[Gauss]	5.18	0.96	3.00	6.00	6.00	26.8
	HMM[Pois]	5.66	0.47	5.00	6.00	6.00	0.00
	HMM[GenPois]	4.19	0.60	3.00	6.00	6.00	61.8

The proposed methods do not need a priori information on the number of different activities performed during the day. The algorithms identify the most appropriate number 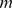 by minimizing AIC and BIC. On average, the simulated days have 3.97 different activities. HMM[GenPois] identifies on average 4.19 followed by HMM[Gauss] with 5.18 (Table 2). HMM[GenPois] identifies the correct number of activities in 61.3% of the days, whereas HMM[Gauss] only in 26.8%. HMM[Pois] does not identify the correct number at all.

Mathematical models often have the disadvantage of being numerically instable or having a long runtime. With the exception of the HMM[GenPois], which was numerically instable in 6.9% of the simulated days, all other presented methods converged for the simulated days. Runtime varied from 0.01 seconds (cutpoint) to 2.0 minutes (HMM[Gauss]) to 14.2 minutes (HMM[GenPois]) on a regular Windows workstation.

## Discussion

This paper investigated the feasibility and the potential advantages of the HMM-based method over the cutpoint approach in identifying the sequence of modes of PA. The results of the simulation study clearly showed the inferiority of the cutpoint method compared to HMM-based approaches. By default the cutpoint method was not able to identify the number of activities performed by a subject. Depending on the specific research question, this may, however, be of particular interest in addition to the correct identification of bouts.

For example, typical recommendations on how much PA children and adolescents need each day suggest 60 minutes (1 hour) or more of physical activity that are age-appropriate, enjoyable and offer variety [29]. While moderate to vigorous activity can be adequately assessed using the cutpoint method, this method leads to a rather rough classification, if one wishes to distinguish the intensities of activities within one activity level, as e.g. between fast walking and slow jogging. Both would be simply assigned to a moderate activity level, whereas HMM-based methods have proven to be much more appropriate for this purpose. The HMM-based method can distinguish those activities and therefore our findings have important implications for the measurement of PA in individuals in free-living conditions for monitoring and surveillance purposes.

Among the HMM-based methods, HMM[Pois] revealed the weakest performance in terms of MCR, bout and activity detection. As anticipated, a simple Poisson distribution cannot accommodate the variance seen in accelerometer data. The results for HMM[GenPois] and HMM[Gauss] were very similar to each other. HMM[Gauss] had a slightly better MCR (1.77% vs. 3.03%), while HMM[GenPois] was better in terms of bout detection (16.1% vs. 12.8%). This is a considerable improvement compared to the cutpoint method, especially if one keeps in mind that a bout is considered as incorrectly identified if the detected bout is just one epoch shorter or longer than the true one. This situation can easily occur at the ‘point of discontinuity’ when the person switches from one activity to the other. HMM[GenPois] also performed better than HMM[Gauss] and HMM[Pois] with regard to the number of correctly identified activities, which may be particularly relevant for the analysis of accelerometer data. According to the present results, HMM[GenPois] outperforms HMM[Gauss] in this respect as reflected by the considerably higher activity detection rate of 61.3% for HMM[GenPois] compared to only 26.8% for HMM[Gauss]. HMM[Pois] did not identify the correct number at all. As the mean of identified activities is greater than the mean number of simulated activities, combined with the fact, that HMM[Pois] was not able to detect the correct number of activities at all, it can be concluded that HMM[Pois] in general overestimates the number of activities.

However, this outperformance of HMM[GenPois] comes at a price, namely runtime and problems with numerical stability. Even with 6 hour days and 15 seconds epochs (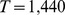), HMM[GenPois] needed seven times more runtime than HMM[Gauss]. As modern accelerometers are becoming increasingly powerful, subjects can be monitored for up to 24 hours a day for 7 or more days at 1–5 seconds epochs. This results in a time series of more than 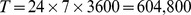 counts, which will dramatically increase the runtime. For small sample sizes this fact can be disregarded and HMM[GenPois] can be used, but in large cohort studies with more than 10,000 subjects, runtime can become an issue and hence HMM[Gauss] may be preferred.

A simulation study was used here to explore the general feasibility and the potential advantages of the presented HMM-based method, as simulated data have the advantage that the ‘truth’ is known for every individual count. That means for example that the activity that generated this count and its true intensity level are known for comparative purposes. Using real-life accelerometer data this information would not be available, even if annotated data with measured oxygen consumption would be at hand. In the present study, the data were generated such that one simulated PA-level was close to a cutpoint to investigate whether HMM-based methods are able to correctly identify PA-levels in such situations. Although the simulation study was especially designed for the comparison of methods when using cutpoints from [9], this does not constitute a limitation to the HMM-based approach presented here since it can be easily adapted to any cutpoints proposed in the literature.

Nevertheless, in a next step, the HMM-based methods have to be applied to real-life accelerometer data, where it will be especially interesting to apply these models to annotated data, where the energy expenditure is known. Another promising future application of the presented method is to use HMMs to estimate 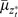 as mean PA-level and use the resulting count estimate in energy prediction equations, as e.g. provided by [30]. The HMM-based methods may lead to improved energy expenditure estimates based on better count estimates.

## Conclusion

HMM-based methods for modeling accelerometer data are a promising extension of the traditional cutpoint method and on the basis of data presented here ought to improve the analysis of PA. While both HMM[GenPois] and HMM[Gauss] methods seem superior to current cutpoint methods, HMM[Gauss] may be more suitable for real-life applications and if estimation of activity levels is not the main focus. HMM[GenPois] should be used if a better activity and bout detection is desired and runtime is not an issue. Despite these encouraging results, both models will have to be applied to real accelerometer data in future studies in order to prove their superiority over traditional cutpoint method in practice.
